# Beyond the double helix: the multifaceted landscape of extracellular DNA in *Staphylococcus aureus* biofilms

**DOI:** 10.3389/fcimb.2024.1400648

**Published:** 2024-06-05

**Authors:** Lucy C. Bowden, Jenny Finlinson, Brooklyn Jones, Bradford K. Berges

**Affiliations:** Department of Microbiology and Molecular Biology, Brigham Young University, Provo, UT, United States

**Keywords:** *Staphylococcus aureus*, biofilm, MRSA, extracellular DNA, bacterial pathogenesis, biofilm formation, biofilm structure

## Abstract

*Staphylococcus aureus* forms biofilms consisting of cells embedded in a matrix made of proteins, polysaccharides, lipids, and extracellular DNA (eDNA). Biofilm-associated infections are difficult to treat and can promote antibiotic resistance, resulting in negative healthcare outcomes. eDNA within the matrix contributes to the stability, growth, and immune-evasive properties of *S. aureus* biofilms. eDNA is released by autolysis, which is mediated by murein hydrolases that access the cell wall via membrane pores formed by holin-like proteins. The eDNA content of *S. aureus* biofilms varies among individual strains and is influenced by environmental conditions, including the presence of antibiotics. eDNA plays an important role in biofilm development and structure by acting as an electrostatic net that facilitates protein-cell and cell-cell interactions. Because of eDNA’s structural importance in biofilms and its ubiquitous presence among *S. aureus* isolates, it is a potential target for therapeutics. Treatment of biofilms with DNase can eradicate or drastically reduce them in size. Additionally, antibodies that target DNABII proteins, which bind to and stabilize eDNA, can also disperse biofilms. This review discusses the recent literature on the release, structure, and function of eDNA in *S. aureus* biofilms, in addition to a discussion of potential avenues for targeting eDNA for biofilm eradication.

## Introduction

*Staphylococcus aureus* is a gram-positive bacterium that is commonly found in the human population, colonizing approximately 30% of humans in the nasal passages (Lister and Horswill, 2014), as well as the skin and gastrointestinal tract (Raineri et al., 2022). *S. aureus* is an important human pathogen since it causes skin infections, bacteremia, osteomyelitis, pneumonia, and endocarditis and leads to nearly 20,000 deaths per year in the US (Kavanagh, 2019). *S. aureus* also exhibits a high level of antibiotic resistance, with many strains demonstrating resistance even to last-resort antibiotics (Guo et al., 2020).

*S. aureus* forms biofilms, which are surface-associated assemblages of bacteria embedded in a self-produced extracellular matrix. These aggregations are an ideal way for bacteria to evade the immune system and to survive in nutrient-poor locations (De La Fuente-Núñez et al., 2013; Yin et al., 2019). Biofilms are of particular concern in medical settings due to the extreme difficulty in treating them (Gebreyohannes et al., 2019). This is due in large part to the limited or delayed diffusion of some antibacterial agents through the biofilm matrix (Singh et al., 2010), as well as the presence of persister cells (Craft et al., 2019) and antibiotic-resistant bacteria (Balcazar et al., 2015). Persister cells are non-dividing cells that exhibit transient antibiotic resistance during antibiotic challenge (Chang et al., 2020). Each of these defense mechanisms make biofilms hard to target with traditional antibiotic regimens.

Biofilm formation in *S. aureus* follows several well-studied steps, including attachment, maturation, and dispersal (Schilcher and Horswill, 2020). First, free-floating *S. aureus* bacteria attach to a surface by hydrophobic interactions, hydrogen bonds, ionic bonds, and/or protein-mediated attachment (Jiang et al., 2021). The bacteria then multiply into a confluent mat of cells (Moormeier and Bayles, 2017). This is followed by a period of exodus where a subpopulation of bacteria is released, allowing for the development of metabolically diverse microcolonies (Grande et al., 2014; Moormeier et al., 2014; Moormeier and Bayles, 2017). The microcolonies grow rapidly, and finally, quorum sensing initiates the dispersal of cells, which begin new biofilms in additional locations (Moormeier and Bayles, 2017).

Biofilms consist of cells surrounded by an extracellular matrix. The composition of this matrix in *S. aureus* is highly strain, time, and condition-dependent (Sugimoto et al., 2018; Lade et al., 2019; Ball et al., 2022). The main components of this self-generated matrix are proteins, polysaccharides, lipids, and extracellular DNA (eDNA) (Karygianni et al., 2020). These components are important attachment and structural components of the biofilm (Moormeier and Bayles, 2017). Extracellular RNA may also be present in *S. aureus* biofilms, where it is hypothesized to associate with eDNA and provide structural support (Chiba et al., 2022). However, the low stability of the RNA molecule and constraints in available extraction protocols have made it difficult to study (Mugunthan et al., 2023). Therefore, this review will focus on eDNA. Although much attention has focused on the protein and polysaccharide biofilm matrix constituents (Hobley et al., 2015; Moormeier and Bayles, 2017), the vital role of eDNA is less-well appreciated. The idea that eDNA was a critical component of the biofilm matrix was first suggested by Whitchurch et al., 2002. They showed that DNase I prevented *Pseudomonas aeruginosa* from forming biofilms, suggesting the importance of eDNA as a structural component (Whitchurch et al., 2002). Since that time, further research has shown that the presence of eDNA in biofilms is nearly universal across bacterial species (Campoccia et al., 2021). In *S. aureus* biofilms, eDNA plays important roles in attachment, structure, and stability. The purpose of this review is to describe recent breakthroughs in our understanding of the characteristics of *S. aureus* eDNA as well as its mechanism of release, roles within the *S. aureus* biofilm, and potential methods of targeting eDNA to disrupt biofilm formation.

## Mechanism of eDNA release

In *S. aureus* biofilms, eDNA is released by lysing a subfraction of the bacterial population in a process that depends on murein hydrolases (Rice et al., 2007). Murein (peptidoglycan) hydrolases cleave covalent bonds in peptidoglycan for a variety of purposes (Vollmer et al., 2008). Autolysis-independent mechanisms of eDNA release have been shown in some species of bacteria such as *E. coli* and *P. aeruginosa*, but not yet in *S. aureus* (Fischer et al., 2014). The process of autolysis relies on several important effector and regulatory proteins. After the murein hydrolases degrade the peptidoglycan barrier, the cell lyses and DNA is released into the surrounding area. The now-extracellular DNA is then able to become part of the biofilm matrix.

### Activity and regulation of the Atl murein hydrolase

**Figure 1:**
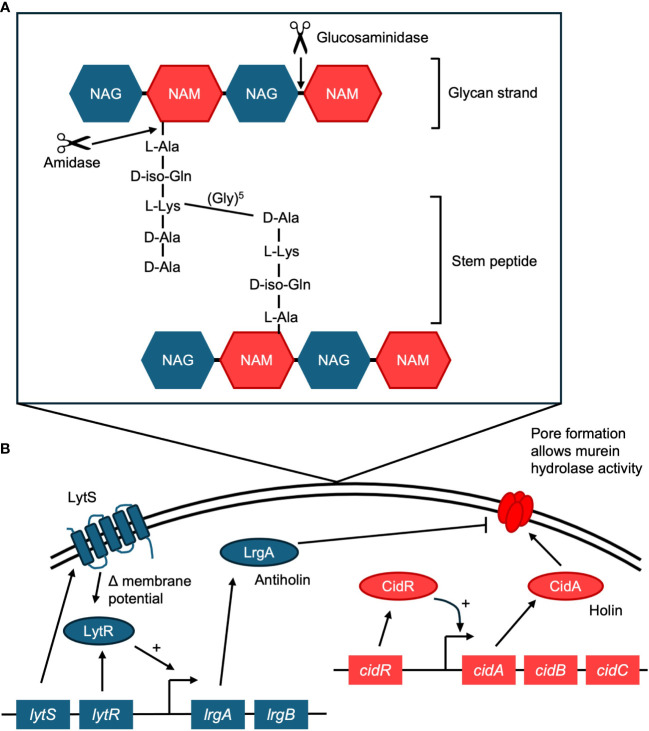
**(A)** Activity of the Atl murein hydrolase. Atl mediates cell lysis in biofilms, resulting in the release of eDNA. Atl is cleaved into two functional enzymes, an amidase and a glucosaminidase. The amidase cleaves the amide bond between the stem peptide and the peptidoglycan (murein) backbone. The glucosaminidase can then cleave the glycan backbone. The glycan strand is made of N-acetyl glucosamine (NAG) and N-acetyl muramic acid (NAM). The stem peptide in S. aureus contains the amino acids L-alanine (L-Ala), D-iso-glutamine (D-iso-Gln), L-lysine (L-Lys), D-alanine (D-Ala), D-alanine (D-Ala). A crosslinking pentaglycine bridge connects adjacent stems. **(B)** Diagram of the regulatory proteins behind the holin/antiholin CidA/LrgA system. CidA oligomerizes to form pores in the membrane, which allows the murein hydrolase to access peptidoglycan, resulting in cell lysis. LrgA acts as an antiholin, preventing CidA from oligomerizing. Cell lysis results in the release of eDNA to the biofilm matrix.

The murein hydrolase, Atl, is a bifunctional enzyme that is cleaved to result in an amidase and a glucosaminidase, and both are required to be catalytically active for *S. aureus* to form a biofilm (Bose et al., 2012) (**Figure 1A**). The amidase cleaves the amide bond between the murein backbone and the stem peptide (Bose et al., 2012), severing the link between the peptide subunit and the muramic acid residues in peptidoglycan (Biswas et al., 2006). This link is one of the critical stress-bearing bonds in the murein netting, and breaking it is a step required for autolysis (Biswas et al., 2006). The activity of the amidase must occur before that of the glucosaminidase, which cannot cleave cross-linked peptidoglycan (Nega et al., 2020). After the amidase has hydrolyzed the cross-peptides, the glucosaminidase cuts the glycan backbone into disaccharides (Nega et al., 2020). This compromises membrane integrity and leads to cell lysis.

Mutations in *atl* result in bacteria that are deficient in both biofilm formation and daughter cell separation (Biswas et al., 2006). Since *S. aureus* can produce minor autolysins such as the *aaa* autolysin, *atl* mutants continue to grow (Biswas et al., 2006). However, they form much weaker biofilms (Ball et al., 2022) with reduced eDNA content (Houston et al., 2011; Bose et al., 2012). When a biofilm formed by an *atl* mutant was treated with DNase I, no significant difference in biomass was found (in contrast to wild-type), indicating that eDNA does not play a significant role in the biofilm of *atl* mutants (Ball et al., 2022). However, it is possible that a loss of the *atl* gene may affect some *S. aureus* isolates differently, particularly those with an *ica*-dependent biofilm phenotype (Houston et al., 2011), as these biofilms may be less reliant on eDNA for structural stability. It should also be noted that not all studies agree that *atl* mutants have substantially lower eDNA, indicating that perhaps other mechanisms of eDNA release are active, or that eDNA release depends on strain- or culture-dependent factors (Dengler et al., 2015; DeFrancesco et al., 2017).

### The role of the CidA/LrgA holin/antiholin system

Cell death and lysis in the *S. aureus* biofilm are controlled by the *cidABC* and *lrgAB* operons (Mann et al., 2009). The product of the *cidA* gene acts as a holin, promoting cell lysis and the release of DNA, and the product of the *lrgA* gene acts as an antiholin, inhibiting lysis (Mann et al., 2009) (**Figure 1B**). Together they regulate cell lysis and murein hydrolase activity, and balanced expression of both is required for normal biofilm maturation (Mann et al., 2009).

### Activity and regulation of the *cidABC* operon

The *cidABC* operon in *S. aureus* regulates cell lysis by having a positive effect on murein hydrolase activity (Rice et al., 2007). In bacteriophage-infected cells, lysis is controlled by an endolysin and a membrane-associated protein known as a holin, which controls the activity of the endolysin, allowing control of the timing of cell lysis (Endres et al., 2021). In *S. aureus* cell lysis, the murein hydrolase acts as the endolysin and CidA as a functional holin (Ranjit et al., 2011; Endres et al., 2021).

CidA oligomerizes and forms pores in the cytoplasmic membrane (Ranjit et al., 2011), which allows the murein hydrolase access to the cell wall, resulting in cell lysis (Windham et al., 2016) (**Figure 1B**). A *cidA* mutant strain produces significantly decreased levels of eDNA (Rice et al., 2007; Mann et al., 2009). A *cidA* mutant is also less susceptible to DNase I treatment than its wild-type counterpart (Rice et al., 2007). Additionally, a study that focused on early biofilm development found that a *cidA* mutant affected biofilm morphology and adherence, possibly by interfering with early attachment and microcolony formation (Rice et al., 2007). The expression of *cidA* is affected by the surrounding environment, with increased expression throughout biofilm development (Grande et al., 2014). Expression of *cidA* is also dependent on local oxygen concentrations (Moormeier et al., 2013).

CidR is a LysR-type regulator of the *cidABC* operon (Yang et al., 2005). CidR increases transcription of *cidA* in the presence of acetic acid, which is produced during the metabolism of glucose (Yang et al., 2005; Patton et al., 2006). However, this increase in transcription is not seen in the presence of other weak acids such as ascorbic acid or pyruvic acid (Patton et al., 2006). Additionally, simply changing the pH by mediating the exchange of protons across the cytoplasmic membrane did not have a large impact on *cidABC* or *lrgAB* expression (Patton et al., 2006). Instead of a pH effect on CidR activation, some specific part of the metabolism of excess glucose, which produces acetic acid, interacts with, and activates CidR, which enhances the transcription of *cidABC* (Patton et al., 2006).

It is possible that in addition to CidA, CidB and CidC also play a role in cell lysis. Comparatively little is known about the role of CidB, but one study found that cell death in a strain lacking the SrrAB two-component system, which represses expression of the *cidABC* operon, was reliant on CidB (Windham et al., 2016). The exact role of CidB in this cell death, however, remains under investigation (Windham et al., 2016). CidC (a pyruvate oxidase) promotes cell death by promoting cytoplasmic acidification and respiratory inhibition by the production of acetate (Thomas et al., 2014). As extracellular pH lowers, acetate becomes acetic acid and can diffuse across the membrane into the cytoplasm (Windham et al., 2016). This accumulation of acetic acid lowers the intracellular pH and over time leads to reactive oxygen species (ROS)-dependent cell damage and death (Windham et al., 2016).

### Activity and regulation of the *lrgAB* operon

The *lrgAB* operon in *S. aureus* regulates cell lysis by having a negative effect on murein hydrolase activity. While CidA acts as a holin, LrgA acts as an antiholin (Ranjit et al., 2011) (**Figure 1B**). Antiholins interact with holins to prevent them from oligomerizing and forming pores in the cell membrane (Ranjit et al., 2011). A *lrgA* mutant produces significantly increased levels of eDNA compared to the wild type, indicating more cell lysis (Mann et al., 2009). A transposon mutant in the *lrgB* gene also results in increased biofilm development and eDNA release, while overexpression of *lrgB* inhibits biofilm formation (Beltrame et al., 2015).

Like *cidA*, expression of the *lgrA* gene is dependent on local oxygen concentrations (Moormeier et al., 2013). While the *cidABC* operon is regulated by CidR, *lrgAB* is activated by the *lytSR* two-component regulatory system (Sharma-Kuinkel et al., 2009). The LytSR system participates in two signal transduction pathways: it senses decreases in membrane potential and induces *lrgA* transcription (Patton et al., 2006; Sharma-Kuinkel et al., 2009), and it also induces *lrgAB* in response to the metabolism of excess glucose (**Figure 1B**).

### Other cell lysis pathways

One study performed a transposon insertion sequencing experiment to identify other genes involved in the process of eDNA release. While they did not find that mutations of *cidA* or *atl* affected eDNA release under their culture conditions, they did find that mutation of the *gdpP* gene resulted in impaired eDNA release and biofilm formation (DeFrancesco et al., 2017). This effect on biofilm formation and biomass may be strain-dependent (Corrigan et al., 2011). The *gdpP* gene is a phosphodiesterase that cleaves cyclic-di-AMP. Deletions of *gdpP* have been previously shown to increase peptidoglycan cross-linking and increase resistance to antibiotics that target the cell envelope. This work suggests a model where a drop in cyclic-di-AMP levels results in compromised cell wall integrity and subsequent cell lysis (DeFrancesco et al., 2017). Substantiating this model, another group found that a mutation in the purine biosynthesis pathway (Δ*purF*) exhibits significant decreases in cyclic-di-AMP levels, decreased biofilm formation, and decreased eDNA levels. Mutants that receive exogenous cyclic-di-AMP produce similar levels of eDNA as the wild-type (Li et al., 2021).

## Other mechanisms that affect the amount of eDNA in the biofilm

### The role of the thermonuclease in biofilm eDNA degradation

The *nuc* gene encodes the staphylococcal thermonuclease, which also plays a role in the amount of eDNA present in a biofilm. The role of *nuc* is to degrade eDNA both to protect *S. aureus* against NETs (Berends et al., 2010) as well as potentially being involved in releasing cells from the biofilm (Moormeier et al., 2014). Strains with low thermonuclease activity have higher biofilm-forming abilities and there is a negative correlation between *nuc* expression levels and the amount of eDNA in the biofilm (Kiedrowski et al., 2011; Yu et al., 2021). A *nuc* mutant has increased eDNA levels and an altered biofilm architecture (Mann et al., 2009).

*Nuc* mutants have been shown to accumulate more high molecular weight eDNA (Kiedrowski et al., 2011), and strains that naturally produce more Nuc may therefore have lower levels of high molecular weight eDNA in the biofilm (Kavanaugh et al., 2019). The evidence presented above has led to the hypothesis that *nuc* is responsible for the degradation of eDNA that results in a release of a subpopulation of cells from the biofilm.

### Inclusion of host/foreign DNA in the *S. aureus* biofilm

Studies of biofilms from other species have speculated that some of the eDNA in an *in vivo* biofilm could be of eukaryotic host origin (Chiang et al., 2013). This host DNA could come from neutrophils and their production of neutrophil extracellular traps (NETs) (Walker et al., 2005). In *P. aeruginosa* biofilms much of the eDNA on the edges of the biofilm (though not its interior) is host DNA rather than bacterial DNA (Alhede et al., 2020). The host eDNA was found to originate from neutrophils, but the majority did not originate from NETs (Alhede et al., 2020). It is unconfirmed whether *S. aureus* biofilms also contain host DNA, but one study found that while the addition of DNase I disrupted cell-cell clumping within the biofilm, the addition of heterologous salmon sperm DNA was able to restore cell-cell interactions in biofilms (Dengler et al., 2015). This result was not confirmed by a separate experiment (Graf et al., 2019). It remains to be seen whether host or other exogenous DNA is an integral part of *S. aureus* biofilms.

### Culture media makeup affects the amount of eDNA in the *S. aureus* biofilm

In *S. aureus* biofilms, size and structure are culture method dependent. For instance, glucose supplementation affects eDNA levels in biofilms, decreasing it in many strains (Sugimoto et al., 2018). However, further research is needed to better understand these results and the mechanism behind them, since other research has shown that glucose results in an increase in the production of CidR (Patton et al., 2006), which increases eDNA release. The presence of glucose has also been found to decrease cyclic-di-AMP levels (DeFrancesco et al., 2017), which results in increased cell lysis. Another common culture media supplement, NaCl, reduced the quantity of eDNA in the extracellular matrix, possibly by inhibiting the association of proteins and eDNA on the bacterial surfaces (Sugimoto et al., 2018).

### Different strains produce different amounts of eDNA

Some studies have sought to classify methicillin-resistant *S. aureus* (MRSA) biofilms as chiefly composed of protein and eDNA (and *ica*-independent) and methicillin-sensitive *S. aureus* (MSSA) biofilms as composed of polysaccharide (*ica*-dependent). However, this description is not true of all strains. Most *S. aureus* isolates possess the *ica* operon, but its expression is tightly regulated and is affected by a variety of environmental conditions (Fitzpatrick et al., 2005). *Ica*-dependent biofilms have at times been found to exhibit lower eDNA quantities than *ica*-independent biofilms (Sugimoto et al., 2018), though it would be simplistic to expect this to be true of every *ica*-dependent strain (Ball et al., 2022). One study of 47 *S. aureus* clinical isolates found that eDNA was present in biofilms from all strains tested regardless of methicillin resistance status (Sugimoto et al., 2018). Likewise, other studies have found that both *ica*-dependent and *ica*-independent biofilm-forming strains are affected by DNase I treatment (Avila-Novoa et al., 2021). This suggests that even biofilms with comparatively lower amounts of eDNA have enough eDNA to provide structural support to the biofilm.

### Presence of subinhibitory antibiotics impacts biofilm development

In some strains of *S. aureus*, subinhibitory levels of beta-lactam antibiotics increase eDNA release as well as biofilm formation (Kaplan et al., 2012a; Mlynek et al., 2016). This is broadly in agreement with the cell lysis methods shown above; damage to the cell wall results in lysis or autolysis, which results in increased eDNA release and therefore increased biofilm formation.

Members of other categories of antibiotics may also be able to increase eDNA release in *S. aureus* biofilms. Subinhibitory levels of clindamycin, a protein synthesis inhibitor, were found to increase biofilm formation and eDNA levels, though this effect may be strain or lineage-specific (Schilcher et al., 2016). Treatment with subinhibitory antibiotics upregulates *atl* expression, potentially increasing eDNA levels within the biofilm (Azzam et al., 2023).

In contrast, one report found that treatment of *S. aureus* biofilms with subinhibitory levels of nisin decreased eDNA content (Andre et al., 2019). Nisin kills bacteria by causing cell wall depolarization and inhibiting peptidoglycan synthesis (Zhou et al., 2014). eDNA release was also decreased after treatment with subinhibitory levels of tunicamycin, a cell wall teichoic acid production inhibitor (Zhu et al., 2018). Further work remains to be done to better understand the role of subinhibitory antibiotics of various classes on eDNA release in the *S. aureus* biofilm. Understanding that the presence of subinhibitory antibiotics could encourage biofilm formation has implications for clinical settings if antibiotic regimens for biofilm-related infections are not followed as directed.

## Characteristics of the eDNA in the *S. aureus* biofilm

The eDNA in *S. aureus* biofilms is composed of genomic DNA released from lysed cells (Mann et al., 2009; Svarcova et al., 2021). Therefore, it is presumed to contain all chromosomally encoded genes (Fischer et al., 2014), and likely also includes extrachromosomal plasmid DNA. Indeed, amplified fragment length polymorphism comparison of eDNA to genomic DNA reveals high similarity between the two in *S. aureus* (Svarcova et al., 2021). This is in contrast to some other bacterial species, which may incorporate eDNA into their biofilm matrices in a sequence-specific manner (Jakubovics et al., 2013). Sequence-specific eDNA incorporation may also occur in mixed-species biofilms containing *S. aureus* (Steinberger and Holden, 2005), due to either differences in eDNA release or post-release DNA modifications.

Matrix eDNA varies in molecular weight and may take on different roles as it is enzymatically or otherwise modified following release. Addition of restriction enzymes to produce fragments <10 kb resulted in near-complete biofilm detachment while fragments of 11–24 kb caused partial detachment (Izano et al., 2008). This suggests that only fragments >11 kb can function as intercellular adhesins (Izano et al., 2008). In support of these results, additional studies have described the presence of high molecular weight eDNA in *S. aureus* biofilms (Kiedrowski et al., 2011; Kavanaugh et al., 2019).

### Conformation of eDNA in the *S. aureus* biofilm

B-DNA is a right-handed DNA helix and is the most common form of DNA. Z-DNA, in contrast, is slightly smaller in diameter (18 angstroms vs. 20 angstroms) and is in a left-handed conformation. Z-form DNA, as well as other non-B conformations, is an unfavorable substrate for DNase I (Ramesh and Brahmachari, 1989). Therefore, the presence of Z-DNA could affect experiments that use DNase I to quantify and understand eDNA.

Z-DNA is abundant in biofilm eDNA for some organisms including *Escherichia coli, Klebsiella pneumoniae*, and *Haemophilus influenzae* (Buzzo et al., 2021). The Z-DNA confers resistance to DNase treatment and reduces neutrophil extracellular trap (NET) function (Buzzo et al., 2021; Goodman and Bakaletz, 2022). Z-DNA is also abundant in mixed-species biofilms that include *S. aureus* (Buzzo et al., 2021). It is currently unknown whether single-species *S. aureus* biofilms contain Z-DNA, but the possibility ought to be taken into consideration, especially considering the general reliance on DNase I to quantify eDNA in biofilms.

### Structure of eDNA in the biofilm matrix

eDNA from the biofilms of diverse species presents a highly structured, lattice-like organization in *in vivo* biofilms (Jurcisek and Bakaletz, 2007; Novotny et al., 2013). This includes clinical samples of *S. aureus* (Idicula et al., 2016). Interestingly, images of these lattice structures also displayed the presence of DNABII proteins, which bind to DNA vertices (Novotny et al., 2013; Idicula et al., 2016). Eventually it was discovered that eDNA-dependent *Staphylococcus epidermidis* biofilms are reliant on Holliday junction (HJ) orthologs at the vertices of the eDNA. A HJ is a four-way branched structure that links two pieces of double-stranded DNA (Song et al., 2022). The eDNA in biofilms is therefore organized into a lattice-like structure with vertices where HJ-stabilizing proteins such as DNABII proteins and RuvA bind. The combination of functional HJ orthologs and binding proteins helps to stabilize the biofilm (Devaraj et al., 2019). HJ equivalents are also found in *in vivo* examples of *E. coli* and non-typable *H. influenzae* biofilms (Devaraj et al., 2019). Given the conservation of this mechanism across three such varied species, and the fact that DNABII proteins are present at DNA vertices in *S. aureus* biofilms from clinical *in vivo* samples (Gustave et al., 2013; Idicula et al., 2016), it is likely that *S. aureus* biofilms also utilize HJ orthologs in eDNA organization.

## Role of eDNA in biofilm development

eDNA plays a vital role in biofilm attachment and early development. Multiple studies have shown that the application of DNase I during early biofilm development results in a reduction in biomass (Mann et al., 2009; Das et al., 2010). Other studies suggest that while eDNA is important for early biofilm formation, its role may not be easily elucidated by DNase I treatment. One group found that in very early biofilm formation (0–8 hours) eDNA is present in the biofilm but protected from nuclease activity until about 4–6 hours into development (Moormeier et al., 2014). Similarly, another report found that DNase I treatment during early biofilm formation did not affect the total number of cells in the biofilm (Grande et al., 2014). Although no difference in biofilm structure was found after two hours of incubation between DNase I and control treatments, differences in biofilm architecture and morphology were noted at 24 and 72 hours, indicating that eDNA is an important component of biofilm structure (Grande et al., 2014). The authors note that some eDNA remained in DNase I-treated biofilms, indicating that some of the eDNA may have been protected from DNase I digestion (Grande et al., 2014). Although results on whether eDNA is vital for early biofilm formation vary, they indicate that it is likely that eDNA plays an important role in biofilm stability under certain conditions.

At one time eDNA was thought to be mainly important for bacterial attachment and early biofilm formation (Houston et al., 2011). However, many studies have found that DNase I can significantly affect and even dissolve older biofilms *in vitro* (Izano et al., 2008; Tetz et al., 2009; Kaplan et al., 2012b; Moormeier et al., 2014; Ball et al., 2022). DNase I treatment was also found to inhibit early (24 hour) biofilm formation in an *in vivo* rabbit model of empyema, a condition marked by pockets of pus collecting in body cavities, particularly the pleural space (Deng et al., 2022).

Less research has been done into the role of eDNA in mature biofilms. One report that investigated *P. aeruginosa* biofilms found that DNase I was capable of reducing biofilm size at 12, 36, and 60 hours, but not at 84 hours (Whitchurch et al., 2002). This could either indicate that eDNA is less important in a mature biofilm, or that it is protected from DNase activity in an older biofilm.

## Interaction of eDNA with other biofilm components

**Figure 2:**
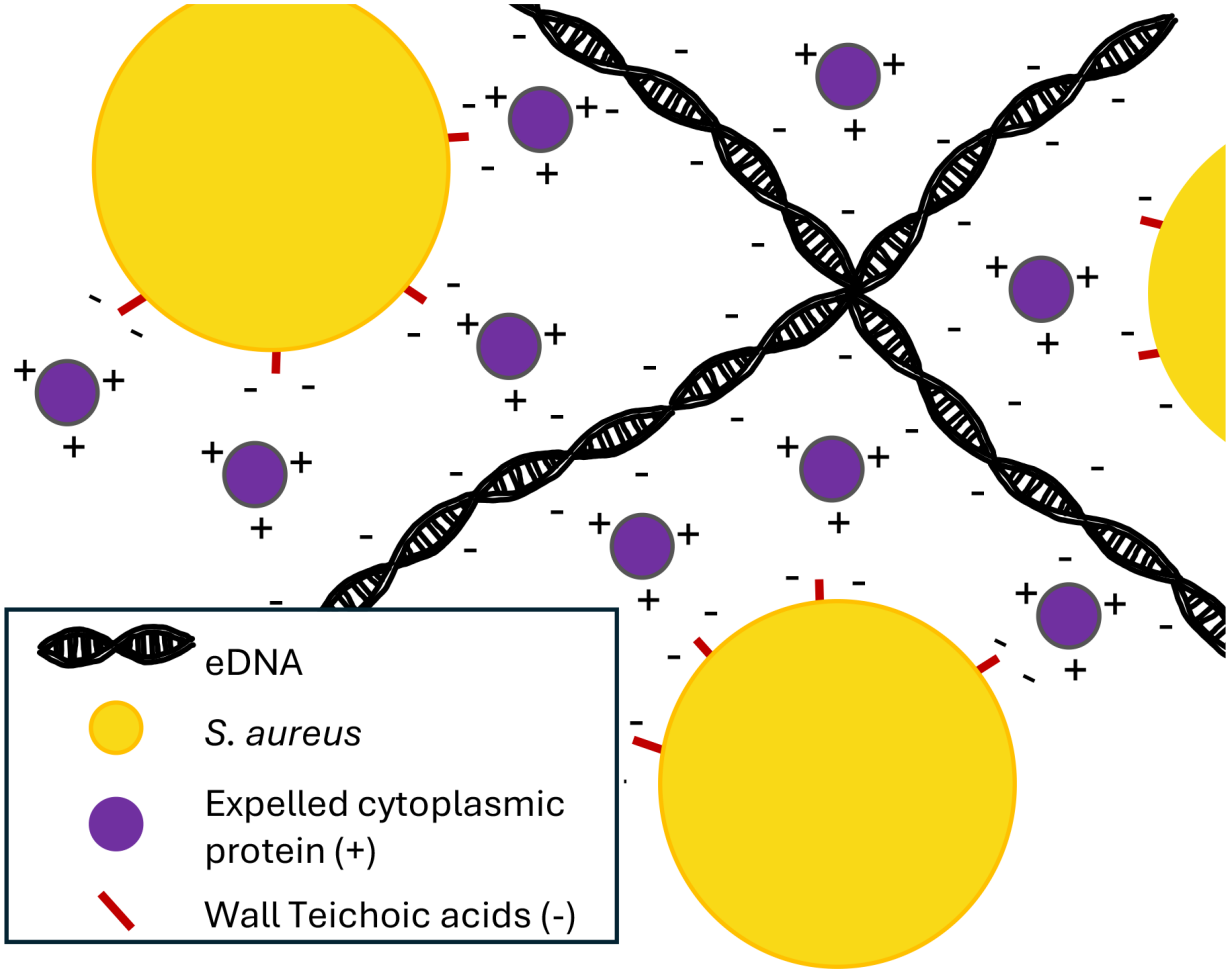
eDNA acts as an electrostatic net. The negatively charged eDNA interacts with positively charged expelled cytoplasmic proteins, which interact with negatively charged proteins on the surface of S. aureus cells, helping to form a cohesive biofilm (Dengler et al., 2015).

One of the key theories of the role of eDNA in the *S. aureus* biofilm is the electrostatic net model. In this model, negatively charged eDNA facilitates cell-cell adhesion by interacting with positively charged matrix proteins, which can interact with negatively charged cell surface molecules such as teichoic acids (**Figure 2**) (Dengler et al., 2015). An additional study found that in the acidic conditions of biofilms, the proteins of the extracellular matrix are strongly positively charged, supporting the model (Graf et al., 2019).

Some other groups have shown that proteins from the cytoplasm of lysed cells may be recycled to act as biofilm matrix proteins (Foulston et al., 2014), binding to eDNA and protecting it from nuclease activity. Such moonlighting proteins associate with cells in biofilms due to the drop in pH found in biofilm interiors (Dengler et al., 2015). These proteins found in the extracellular matrix have a high isoelectric point and therefore in the acidic milieu of the interior of a biofilm will be strongly positively charged (Graf et al., 2019). This result corroborates those found by Dengler et al., suggesting that the negatively charged eDNA can act as an electrostatic net, connecting positively charged proteins and anionic cell surfaces (Dengler et al., 2015). Furthering this paradigm, work by Kavanaugh et al. confirmed the presence of these positively charged proteins in addition to showing that membrane-attached lipoproteins can interact with matrix eDNA. These lipoproteins can function as anchors between matrix eDNA and cell surfaces (Kavanaugh et al., 2019).

Interestingly, while it is well known that the interior of biofilms is acidic, one group found that in *P. aeruginosa*, eDNA may be partially responsible for the acidification of the biofilm (Wilton et al., 2016), and a similar mechanism may be in play for *S. aureus*. The acidic environment is then ideal for eDNA to act as a stabilizing electrostatic net.

eDNA is known to associate with cells in a biofilm in very specific ways. A study by Dengler et al. degraded proteins in the matrix of *S. aureus* biofilms and observed that eDNA was freed from the cell surface. This indicates that the specific methods by which eDNA interacts with biofilm cells depend on the proteins within the matrix (Dengler et al., 2015). Such a claim would suggest that eDNA interactions are complex and largely not understood and can change depending on the protein composition of the biofilm (Dengler et al., 2015). Nevertheless, it is suggested that eDNA serves to hold cells in place by creating a web of interactions with the matrix proteins (Dengler et al., 2015).

There have been several studies into the interactions of eDNA with specific biofilm matrix components. Beta toxin is a neutral sphingomyelinase that belongs to the DNase I superfamily (Huseby et al., 2007). A study by Huseby et al (Huseby et al., 2010). showed that rather than simply degrading eDNA, co-incubation of beta toxin and DNA resulted in the formation of a precipitate of beta toxin oligomers. Thus, beta toxin cross-links in the presence of DNA, forming an insoluble matrix that stimulated biofilm formation *in vivo*. This points to a molecular mechanism for a structural framework for some staphylococci biofilms, but many strains of *S. aureus* do not express beta toxin, indicating that many other mechanisms must be at work (Huseby et al., 2010). This research is also similar to studies in other bacterial species showing that proteins with DNA-binding activity may be important biofilm matrix components (Kavanaugh et al., 2019).

However, not all proteins with DNA-binding activity appear to contribute to biofilm structure or formation in all strains (Mackey-Lawrence et al., 2009). IsaB is a protein that was discovered to have DNA-binding capabilities but its deletion did not result in changes to biofilm biomass (Mackey-Lawrence et al., 2009). Further studies determined that in a different strain that had previously reported higher levels of secreted IsaB, a 2-fold reduction in biofilm formation was found in an *isaB* mutant (Kavanaugh et al., 2019). Further, they determined that deletion of both IsaB and another DNA-binding protein, Eap resulted in a reduction of eDNA, suggesting that in some cases eDNA-binding proteins may act redundantly to bind eDNA in the biofilm matrix (Kavanaugh et al., 2019).

*S. aureus* also produces phenol-soluble modulins (PSMs) that are involved in biofilm structure and dissemination (Schilcher and Horswill, 2020). PSMs can disperse biofilms but they can exist in a polymerized, amyloid-like form in stable biofilms (Zheng et al., 2018). PSMs were found to attach to eDNA and were found in some cases to provide resistance against DNase digestion (Zheng et al., 2018), though earlier research had postulated that the presence of eDNA promoted amyloid formation by PSMs (Schwartz et al., 2016).

In addition to binding to proteins to stabilize the biofilm matrix, eDNA interacts with the poly-*N*-acetylglucosamine (PNAG) polysaccharide to stabilize the biofilm (Mlynek et al., 2020). It was once thought that *S. aureus* produced one of two possible biofilm morphologies based on either PNAG or eDNA/protein. However, these morphologies are not mutually exclusive, as discussed above, and isolates that produce large amounts of polysaccharide also produce eDNA (Sugimoto et al., 2018). In biofilms, PNAG carries a net positive charge and thus may directly interact with eDNA as part of the electrostatic net model (Mlynek et al., 2020). Due to an expanding understanding of *S. aureus* biofilm matrix composition, the relationship and interaction between PNAG and eDNA is an area of active research. Further research into eDNA interactions with polysaccharides needs to be done to conclude whether an interaction between them is a widespread phenomenon important to biofilm structure.

## Other roles of eDNA

The mechanical strength and structure of a biofilm is affected by the amount of eDNA present. Biofilms are both viscous (resistant to flow) and elastic (returning to their original shape and size when force is removed) (Peterson et al., 2015). These properties help the bacteria within the matrix to survive various stresses such as fluid flow or mechanical detachment. In one study, biofilms of *S. aureus* and several other species were mechanically deformed, and the stress relaxation was quantified. Principle component analysis revealed that eDNA contributes to viscoelastic relaxation, the ability of the biofilm to rebound after stress is placed upon it (Peterson et al., 2013). Even relatively small changes in biofilm viscoelasticity may impact a biofilm’s resistance to phagocytosis as well as the time required for effective phagocytosis (Wells et al., 2023). From a mechanical standpoint, eDNA is considered to be an effective construction material, participating not only in biofilm structure but also in biofilm remodeling. This is due to environmental mechanical forces such as shear. Biofilm mechanics in response to shear and compressive forces were found to depend on the concentration of eDNA and the eDNA-to-cell ratio (Lysik et al., 2022).

In addition to its role as a mechanical stabilizer, in some bacteria eDNA also acts as a mechanism for horizontal gene transfer (Panlilio and Rice, 2021). Although the *S. aureus* genome has competence genes, *S. aureus* displays natural competence only under certain conditions. One study has found that in *S. aureus* biofilms, horizontal gene transfer of the SCC*mec* gene could occur between heat-killed cells and living cells. These results could suggest the existence of horizontal gene transfer involving eDNA in *S. aureus* biofilms in other environments (Maree et al., 2022). Additionally, microaerobic conditions may induce natural competence in *S. aureus* (Feng et al., 2023). Oxygen-poor microenvironments can be found in biofilms, which may provide the proper environment for horizontal gene transfer in the form of transformation to take place.

Not all of the roles of eDNA are helpful to the biofilm—it can also act as a pathogen-associated molecular pattern (PAMP). Bacterial eDNA can be recognized by the innate immune system by toll-like receptors, particularly TLR9, which is triggered upon phagocytosis of eDNA (Knuefermann et al., 2007; Campoccia et al., 2023). One group found that the treatment of *P. aeruginosa* biofilms with DNase I reduced the ability of the biofilm to upregulate neutrophil activation markers and reduced the release of neutrophil proinflammatory cytokines (Fuxman Bass et al., 2010). However, another study of *in vivo S. aureus* showed that these biofilms were capable of evading detection by both TLR9 and TLR2 (Thurlow et al., 2011).

## eDNA and biofilm eradication

A better understanding of the role of eDNA in biofilms can lead to the development of treatments for biofilm-related infections that target eDNA (**Table 1**). DNase treatment has previously been found to prevent biofilm formation (Mann et al., 2009), but results have been mixed as to when during biofilm development it may be effective, or whether it is effective at all (Grande et al., 2014; Moormeier et al., 2014). This is possibly due to either proteins that protect eDNA from DNase I treatment (Grande et al., 2014), or the potential accumulation of Z-form DNA (Ramesh and Brahmachari, 1989; Buzzo et al., 2021). As discussed above, both conditions result in eDNA that is not a favorable substrate for DNase I treatment.

**Table 1:** Treatments targeting eDNA to eradicate biofilm-related infections.

Treatment	Reference
DNase + tissue plasminogen activator	(Rahman et al., 2011; Kacprzak et al., 2013; Piccolo et al., 2015; Mehta et al., 2016)
DNase + antibiotic therapy	(Li et al., 2023)
DNase pretreatment of medical devices	(Aktan et al., 2022)
Antibody treatment against HU protein	(Gustave et al., 2013; Idicula et al., 2016; Kurbatfinski et al., 2022)
Antibody treatment against HU protein + antibiotic therapy	(Estelles et al., 2016; Kurbatfinski et al., 2022; Rogers et al., 2022)

However, DNase may be a possible treatment for some applications. DNase I has shown promise in empyema models both *in vitro* and *in vivo* (Deng et al., 2022). When DNase was used in conjunction with tissue plasminogen activator, the combination therapy resulted in undetectable S. aureus levels in about 90% of patients without the need for surgery (Piccolo et al., 2015; Mehta et al., 2016), as well as improving pus viscosity (Kacprzak et al., 2013) and pleural drainage (Rahman et al., 2011). DNase may be a potential therapeutic for other disease models. Potential avenues include the administration of DNase in combination with antibiotic therapy (Li et al., 2023) as well as DNase pre-treatment of medical implants (Aktan et al., 2022).

Not all therapeutic anti-biofilm efforts revolve around the application of DNase. The DNABII family of proteins is ubiquitously expressed in all eubacterial species (Goodman and Bakaletz, 2022). They are small, basic proteins that bind to bent DNA and have been found to contribute greatly to the end structure of eDNA in biofilms of many pathogens including non-typable *H. influenzae* (Goodman et al., 2011)*, E. coli* (Devaraj et al., 2015), and *Streptococcus gordonii* (Rocco et al., 2017), by acting as a binding agent at the vertices of the eDNA (Goodman and Bakaletz, 2022). DNABII proteins are also present in the biofilms of *S. aureus* (Goodman et al., 2011).

Both of the two members of the DNABII protein family, integration host factor (IHF) and histone-like protein (HU) do not have any known homologs in mammalian species (Rogers et al., 2022). *S. aureus* does not code for the integration host factor, but does contain the gene for the histone-like protein (Rogers et al., 2022). HU binds to and bends double-stranded DNA in a non-sequence-specific manner, but it has a high affinity for highly structured dsDNA, such as Holliday junctions (Goodman et al., 2011) and DNA bent at various angles (Swinger and Rice, 2004). Since biofilm eDNA contains structural Holliday junction orthologs (Devaraj et al., 2019), it is unsurprising that proteins such as IHF and HU are important to biofilm stability.

It has been proposed that disruption or depletion of DNABII proteins is a potential therapeutic treatment against biofilms (Novotny et al., 2016; Rocco et al., 2017; Goodman and Bakaletz, 2022), and preclinical *ex vivo* data suggest that it could be effective against multiple pathogens, including *S. aureus* (Gustave et al., 2013; Idicula et al., 2016). Treatment of *in vitro S. aureus* biofilms with a monoclonal antibody treatment against HU resulted in a dose- and time-dependent disruption of the biofilm within about 15 minutes, continuing to increase until a 60-minute time point (Kurbatfinski et al., 2022).

This treatment also resulted in the released cells from the biofilm being more susceptible to antibiotic treatment (Kurbatfinski et al., 2022). This increase in antibiotic susceptibility could be due to either increased exposure of cells within the biofilm as the structure was degraded, and/or due to increased release of bacteria from the biofilm into the planktonic state (Goodman et al., 2011). In a mouse implant infection model using *S. aureus*, the addition of anti-DNABII antibodies and daptomycin significantly reduced both biofilm and planktonic bacteria compared to the administration of daptomycin alone (Estelles et al., 2016).

**Figure 3:**
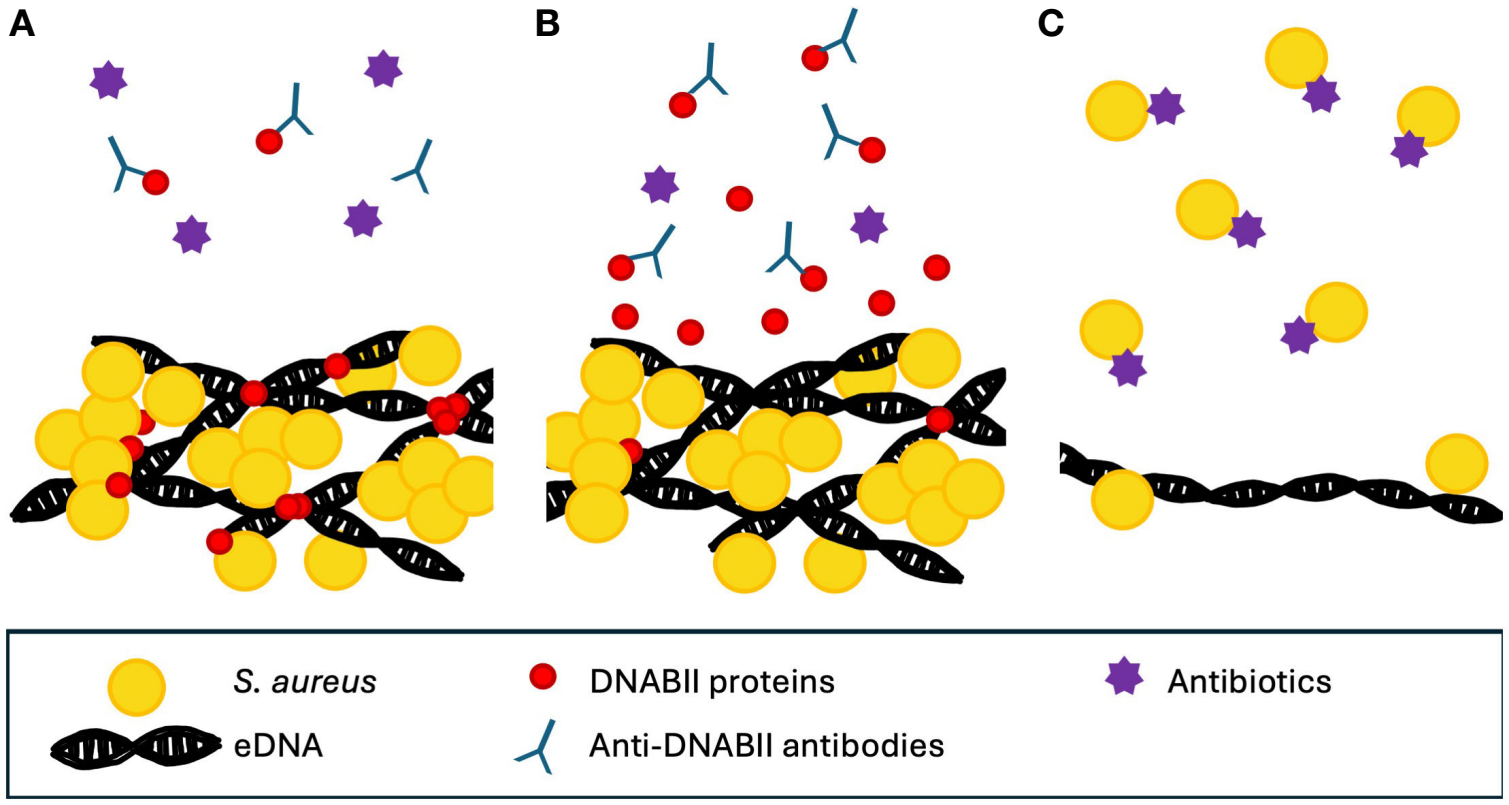
Proposed mechanism for the action of anti-DNABII antibodies. **(A)** DNABII proteins bind to Holliday junction orthologs. The addition of anti-DNABII antibodies removes free DNABII from outside the biofilm. **(B)** This results in diffusion of DNABII proteins away from the biofilm matrix. **(C)** The loss of DNABII proteins compromises the integrity of the biofilm, resulting in release of cells which are then more accessible to antibiotics (Brockson et al., 2014). Note that conceptual figure is not drawn to scale.

One potential mechanism for the ability of anti-DNABII-antibody-induced biofilm collapse suggests that the antibodies bind to free DNABII proteins in the biofilm environment. This causes an equilibrium shift between free and eDNA-bound DNABII proteins, resulting in a release of DNABII proteins from the biofilm. The loss of the DNABII proteins results in biofilm structural collapse (Brockson et al., 2014) (**Figure 3**). This collapse releases bacteria into the surrounding environment, rendering them more susceptible both to antibiotic treatment and to clearance by the host immune system (Rogers et al., 2022).

## Concluding remarks

eDNA has a pivotal role in the development and architecture of *S. aureus* biofilms. The eDNA found in *S. aureus* biofilms is composed of DNA released from lysed cells during biofilm formation. This cell lysis is facilitated by the murein hydrolase, Atl, which is regulated by the holin/antiholin CidA/LrgA system. The process of cell lysis is also potentially influenced by factors such as cyclic-di-AMP levels and other hydrolases. The release of eDNA is contingent upon various factors including culture conditions, individual strain characteristics, and the presence of subinhibitory antibiotics.

Functioning as a crucial structural component within biofilms, eDNA significantly impacts biofilm adhesion, as evidenced by the substantial effects of DNase I on biofilm size and attachment. Acting as an electrostatic net, eDNA binds proteins together, facilitating cell-cell connections. Due to its strong negative charge, it can interact with positively charged proteins in the biofilm matrix, which in turn interact with negatively charged cell surface molecules. This results in a strong adhesion between various biofilm components, which protect the biofilm from removal agents.

The ubiquity of eDNA in *S. aureus* isolates suggests its potential as a general target for biofilm eradication. DNase, commonly used to study the effects of eDNA in biofilms, has potential as a therapeutic agent, especially in combination with other therapies. However, its efficacy may be limited by mechanisms that protect eDNA from DNase activity. An alternative approach involves targeting the DNABII family of proteins that bind to and stabilize bent DNA. This method demonstrates versatility against a wide variety of biofilm-forming pathogens and enhances the effectiveness of concurrent antibiotic treatments.

Despite these improved insights into the role of eDNA in *S. aureus* biofilms, there are many areas that warrant further investigation. One critical aspect is an improved understanding of the mechanisms behind the observed variations in the outcomes of DNase I treatment of *S. aureus* biofilms. Since DNase I is one of the most common methods of studying eDNA production in *S. aureus* biofilms, the limitations of this approach influence our current understanding of eDNA.

Another area of study that requires more in-depth study is the impact of subinhibitory antibiotics on eDNA production, as well as their influence on biofilm formation and stability. Understanding these interactions is important in the development of effective strategies for biofilm-associated infection management. Additionally, more exploration of the relationship between glucose metabolism and eDNA production is warranted. Glucose is widely used as an additive in culture media to increase biofilm formation, and also has relevance to research on diabetes-associated infections. Furthermore, the investigation into the proteins that bind eDNA opens avenues for potential therapeutic interventions. Continued research in this domain may reveal novel approaches for treating biofilm-associated infections. This area of research includes exploring the viability of targeting the DNABII family of proteins in actual patients. These unresolved aspects of eDNA in biofilm formation emphasize the ongoing challenges and opportunities behind understanding *S. aureus* biofilms.

eDNA plays an indispensable role as a structural component of *S. aureus* biofilms. This makes it a promising target for treatment strategies against a pathogen associated with significant morbidity and mortality. The exploration of innovative approaches to manipulate eDNA holds potential for advancing biofilm eradication efforts and improving therapeutic outcomes.

## Author contributions

LB: Writing – review & editing, Writing – original draft, Supervision, Conceptualization. JF: Writing – original draft. BJ: Writing – original draft. BB: Writing – review & editing, Project administration, Supervision.
